# Bacteria from extreme environments as candidate bioinoculants for drought tolerance in legumes: a critical appraisal of mechanisms and evidence

**DOI:** 10.3389/fpls.2026.1944380

**Published:** 2026-10-20

**Authors:** Deisy Johana Cuellar Lopez, Rodrigo Mora-Sanhueza, Ricardo Tighe-Neira, Braulio Soto-Cerda, Marjorie Reyes-Díaz, Kattia Núñez-Montero, Patricio Arce-Johnson, Dimas Mendes Ribeiro, Claudio Inostroza-Blancheteau

**Affiliations:** 1 Programa de Doctorado en Ciencias Agropecuarias, Facultad de Recursos Naturales, Universidad Católica de Temuco, Temuco, Chile; 2 Programa de Doctorado en Fisiología Vegetal, Departamento de Biologia Vegetal, Universidade Federal de Viçosa, Viçosa, MG, Brazil; 3 Laboratorio de Fisiología y Biotecnología Vegetal, Departamento de Ciencias Agropecuarias y Acuícolas, Facultad de Recursos Naturales, Universidad Católica de Temuco, Temuco, Chile; 4 Núcleo de Investigación en Producción Alimentaria, Facultad de Recursos Naturales, Universidad Católica de Temuco, Temuco, Chile; 5 Agencia Nacional de Investigación y Desarrollo - Millennium Science Initiative Program, Millennium Nucleus in Data Science for Plant Resilience (Phytolearning), Santiago, Chile; 6 Laboratorio de Ecofisiología Molecular y Funcional de Plantas, Departamento de Ciencias Químicas y Recursos Naturales, Facultad de Ingeniería y Ciencias, Universidad de La Frontera, Temuco, Chile; 7 Center of Plant, Soil Interaction and Natural Resources Biotechnology, Scientific and Technological Bioresource Nucleus (BIOREN-UFRO), Universidad de La Frontera, Temuco, Chile; 8 Instituto de Ciencias Aplicadas, Facultad de Ingeniería, Universidad Autónoma de Chile, Santiago, Chile

**Keywords:** bioinoculants, drought stress, evidence classification, extremophiles, extremotolerant bacteria, legumes, plant growth-promoting bacteria

## Abstract

Drought is one of the major constraints limiting legume productivity, impairing plant growth, biological nitrogen fixation, and seed nutritional quality. The increasing frequency and intensity of water scarcity have intensified the search for sustainable strategies to improve crop resilience. Bacteria isolated from extreme environments have been proposed as candidate bioinoculants because adaptations to desiccation, salinity, or extreme temperatures may provide traits relevant to plant performance under water deficit. However, most of these isolates are extremotolerant rather than extremophilic sensu stricto, and much of the supporting evidence derives from stresses other than drought. The objective of this review is to synthesize current knowledge on the mechanisms through which extremophilic and extremotolerant bacteria may contribute to drought tolerance in legumes, using explicit operational definitions for these microbial categories and for drought, and a four-tier evidence classification applied at the level of individual studies. Candidate mechanisms include exopolysaccharide- and biofilm-mediated rhizosphere water retention, modulation of root architecture and auxin/ethylene balance through ACC deaminase activity, osmolyte accumulation, antioxidant responses, nutrient mobilization, maintenance of nodulation and biological nitrogen fixation, and regulation of host stress-responsive genes. Direct evidence, defined as extremophilic or extremotolerant strains evaluated in legumes under soil water deficit, is restricted to a small number of studies, whereas a large proportion of the literature relies on salinity, heat, or PEG-induced osmotic stress, non-legume hosts, genomic potential, or *in vitro* assays. We also discuss constraints that may limit the use of these bacteria, including rhizosphere colonization, persistence, and competitiveness against native microbiota under non-extreme conditions. Current findings provide plausible mechanisms and proof-of-concept results, but they do not yet demonstrate reliable field performance of extremophile-derived inoculants in legume production. Strain-level characterization, standardized drought protocols, and multi-environment field validation are required to establish their agronomic value.

## 1 Introduction

Legumes are essential for global food security and human nutrition because they produce protein-rich seeds with high nutritional value (Goyal et al., 2021; Singh et al., 2022a). A distinctive feature of this crop group is its ability to establish symbiotic associations with rhizobia, enabling biological nitrogen fixation, reducing the need for synthetic nitrogen fertilizers, and improving soil fertility (Losa et al., 2021; Lisciani et al., 2024). Despite this relevance, legumes are particularly sensitive to drought. Water deficit affects vegetative growth, photosynthesis, nutrient acquisition, hormonal balance, and biological nitrogen fixation, resulting in substantial yield losses (Goyal et al., 2021; Khatun et al., 2021). A global meta-analysis further reported marked differences in drought sensitivity among legume species. Under water reductions exceeding 65%, yield declined by 40% in faba bean (*Vicia faba*), compared with 21.7% in lentil (*Lens culinaris*) and 28.6% in groundnut (*Arachis hypogaea*). At 60–65% water reduction, common bean (*Phaseolus vulgaris*) showed the greatest yield loss (60.8%), whereas soybean (*Glycine max*) was the least affected (28.0%) (Daryanto et al., 2015). Beyond reducing productivity, drought also alters seed nutritional quality by affecting the accumulation of proteins, amino acids, minerals, carbohydrates, lipids, and other bioactive compounds. These responses vary according to the species, genotype, and timing of stress exposure (Machado et al., 2025). Drought also alters soil properties and microbial communities, disrupting nutrient cycling and plant–soil–microbiome interactions (Gao et al., 2022; Cordero et al., 2023).

Although conventional strategies, including genetic improvement and the selection of drought-tolerant cultivars, have contributed to enhancing legume adaptation, their implementation remains slow because drought tolerance is a complex trait and strongly influenced by genotype × environment interactions (Khatun et al., 2021; Acuña-Rodríguez et al., 2019; Siddiqui et al., 2015). Therefore, complementary and sustainable strategies are needed to enhance the capacity of legumes to maintain growth, productivity, and biological functions under increasingly frequent and severe drought events. Among these approaches, bacteria isolated from extreme environments have been proposed as candidate bioinoculants. Unlike most conventional plant growth-promoting bacteria (PGPB), which are typically isolated from agricultural soils, these microorganisms originate from habitats characterized by desiccation, salinity, extreme temperatures, radiation, heavy metals, or extreme pH (Sibanda et al., 2025). Adaptation to such conditions has been associated with the production of osmoprotectants, antioxidants, exopolysaccharides, secondary metabolites, stress-stable enzymes, and efficient molecular repair systems (Asghari et al., 2020; Sibanda et al., 2025). These traits support microbial activity under stress and have been proposed to contribute to nutrient availability, hormonal regulation, and protection against oxidative damage in the host (Gaete et al., 2020; Tiwari et al., 2024). Conventional PGPB can express similar functions, including the production of phytohormones, ACC deaminase, and compatible osmolytes, but their performance may decline under highly fluctuating environmental conditions; bacteria adapted to extreme habitats are hypothesized, although not yet demonstrated, to maintain these functions under more severe stress (Pishchik et al., 2024; Sheoran et al., 2025). For example, halophilic strains of *Stenotrophomonas* and *Exiguobacterium* isolated from extreme environments increased soybean germination and root growth under 250 mM NaCl (Santos et al., 2023), and nutrient-solubilizing extremophiles have been proposed as bioinoculants for stress-prone agricultural systems (Bharat et al., 2024). Because functional traits and plant growth-promoting performance vary substantially among strains within these genera, such effects cannot be generalized at the genus level.

Despite their promising potential, the microbial diversity of extreme environments remains largely unexplored, and the mechanisms underlying plant–extremophile interactions are still poorly understood. Moreover, the application of extremophilic bacteria in agriculture remains incipient because research has primarily focused on industrial biotechnology, driven by the exceptional stability of extremozymes such as the Taq DNA polymerase from *Thermus aquaticus*. Consequently, studies exploring their use as bioinoculants under drought conditions are still limited (Benítez‐Mateos and Paradisi, 2023; Chia et al., 2024). Furthermore, much of the available evidence is derived from non-legume species, salinity or osmotic stress assays, or microorganisms without a confirmed extremophilic origin, limiting our understanding of legume–extremophile interactions under actual drought conditions. Therefore, the objective of this review is to synthesize current knowledge on the mechanisms through which extremophilic and extremotolerant bacteria may contribute to drought tolerance in legumes. Rather than assuming that all bacteria from extreme environments confer drought tolerance, this review first defines the microbial categories and the drought criteria used throughout the text (Section 2.1; **Figure 1**), then classifies the evidence for each mechanism at the level of individual studies (**Table 1**), distinguishing direct evidence obtained in legumes under actual drought from adjacent, non-legume, and mechanistic evidence, and finally discusses the ecological and practical constraints that may limit the use of these bacteria as inoculants (Section 7). This approach identifies the mechanisms supported by direct evidence, highlights current knowledge gaps, and outlines priorities for future research.

**Figure 1:**
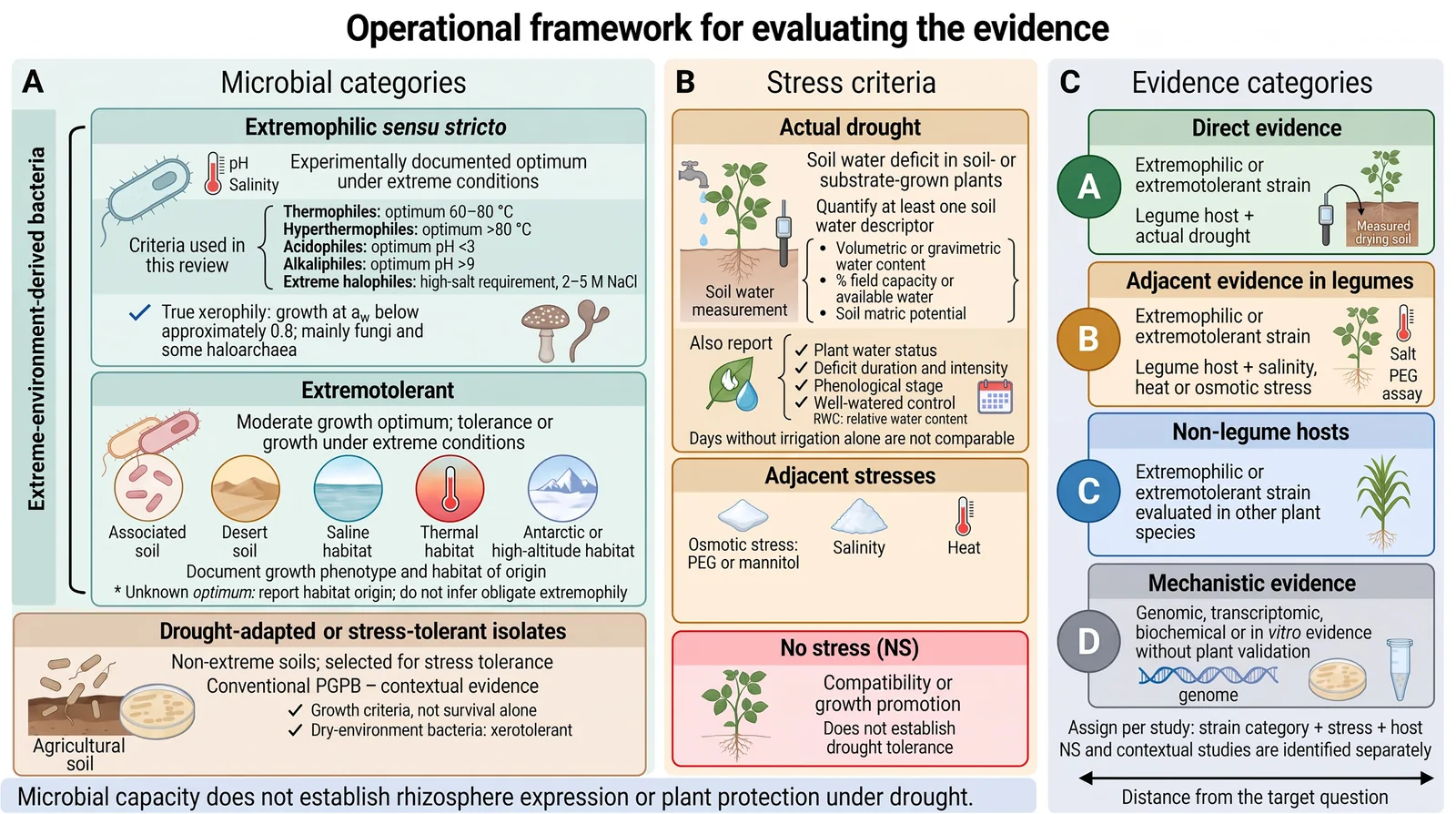
Operational framework used in this review. **(A)** Microbial categories. Extremophilic strains require an extreme condition for optimal growth; extremotolerant strains grow optimally under moderate conditions but tolerate, or grow under, extreme conditions, or were isolated from a documented extreme habitat. Together, they constitute extreme-environment-derived bacteria. Drought-adapted or stress-tolerant isolates from non-extreme soils are treated as contextual evidence. True xerophiles, able to grow below a water activity of approximately 0.8, are almost exclusively fungi and some haloarchaea; bacteria from dry environments are therefore considered xerotolerant. **(B)** Stress criteria. Actual drought is a soil water deficit documented by at least one quantitative descriptor of soil water status; PEG or mannitol osmotic stress, salinity, and heat are adjacent stresses and are not considered evidence of drought tolerance. **(C)** Evidence categories, assigned to each study according to strain category, stress imposed, and host. Microbial capacity documented in genomes or *in vitro* (category D) is not equivalent to trait expression in the rhizosphere or to agronomic efficacy.

**Table 1:** Categories of extremophilic and extremotolerant bacteria evaluated as candidate bioinoculants for drought tolerance, representative strains, documented traits, and evidence category assigned to each study.

Category (operational criterion)	Representative strains	Documented traits	Evidence by study: host; stress; category
Xerotolerant: isolated from arid or hyperarid soils, desert plants, or chasmophytes	*B. paralicheniformis* L38, *Pseudomonas* sp. LN75, *Enterobacter hormaechei* subsp. *xiangfangensis* LJ89, *B. paramycoides* L17, and *Micrococcus luteus* LA9 (chasmophytic *Chenopodium*); *Mesorhizobium ciceri* RZ-11, *B. subtilis* RP-01, and *B. mojavensis* RS-14 (Thal Desert soil); *B. badius* G8-6, *B. paralicheniformis* G11-5, and *Stenotrophomonas pavanii* G12-4 (arid soils, western Rajasthan); *Bacillus* spp. from the Atacama Desert; *Arthrobacter* sp. Helios	Compatible solutes, EPS and biofilm, IAA, ACC deaminase, P, K, and Zn solubilization, siderophores	Chickpea; water stress, greenhouse and field; A (Das et al., 2024) Chickpea; 55–95% field capacity; A (Aulakh et al., 2020) Clusterbean; drought in pots and rainfed field; A (Kasana et al., 2023) Lettuce; drought; C (Santander et al., 2024) Culture; PEG-induced osmotic stress, transcriptome; D (Hernández-Fernández et al., 2022)
Halotolerant or halophilic: isolated from saline or saline-arid environments	*B. tequilensis*; halophilic ACC deaminase-positive isolates; halophilic *Stenotrophomonas* and *Exiguobacterium* strains; halotolerant durum-wheat rhizobacteria; halophilic and halotolerant crop-rhizosphere isolates	Osmolytes, EPS and biofilm, ACC deaminase, IAA, P solubilization (*pqqE*), extremozymes	Chickpea; salinity; B (Qurashi and Sabri, 2012; Nagaraju et al., 2021; Haroon et al., 2023) Soybean; salinity; B (Santos et al., 2023) Durum wheat; drought; C (Albdaiwi et al., 2019) Culture; enzymes and compatible solutes; D (Reang et al., 2024)
Thermophilic or thermotolerant: isolated from hot springs	*B. paralicheniformis* CamBx3; *B. haynesii* CamB6; *B. subtilis* BHUJP-H1, *Bacillus* sp. BHUJP-H2, and *B. licheniformis* BHUJP-H3 (Chumathang hot spring); *B. cereus* SA1; *Bacillus* spp. from Jakrem hot spring	Thermostable enzymes, chaperones (*dnaK*, *dnaJ*), Mrp antiporters, thermostable EPS with antioxidant and water-holding capacity	Mungbean; no stress; NS (Verma et al., 2018) Soybean; heat; B (Khan et al., 2020b) Tomato; heat; C (Khan et al., 2020a) Genome and EPS characterization; D (Narsing Rao et al., 2024; Díaz-Cornejo et al., 2023) PGP traits; D (Arora and Panosyan, 2019)
Psychrotolerant, high-altitude, or Antarctic origin	*Rhizobium* sp. QHCD11 (Qinghai–Tibet Plateau); Antarctic *Pseudomonas* sp. ATCC PTA-122608; *Pseudomonas* sp. PGERs17 (north-western Himalaya); *Pseudomonas* sp. TmR5a and *Curtobacterium* sp. BmP22c (Andes and Patagonia)	Cold-active enzymes, ice recrystallization inhibition, ACC deaminase, IAA	Faba bean; drought; A (Li et al., 2023) Soybean; no stress; NS (Garcia et al., 2021) Wheat; low temperature; C (Mishra et al., 2008) Tomato; cold stress; C (Vega-Celedón et al., 2021) Review (Chauhan et al., 2023)
Acidotolerant or acidophilic	*Acidithiobacillus thiooxidans* and *A. ferrooxidans*, alone or with *B. megaterium* and *P. fluorescens*; acidophilic P-solubilizing bacteria; *B. pumilus* ES4	Rock phosphate and K-rock solubilization, IAA, siderophores	Rock incubation *in vitro*; D (Silva et al., 2020) *In vitro* solubilization; D (do Carmo et al., 2026) Wheat; C (Ahmad et al., 2024) Mine tailings, non-legume host; C (de‐Bashan et al., 2010)
Polyextremophilic	*Natranaerobius thermophilus*	Osmotic homeostasis, ion transport, heat-shock proteins	Mechanistic only; D (Tiwari et al., 2024)
Contextual: drought-adapted isolates from non-extreme soils or host tissues	*Ochrobactrum pseudogrignonense* RJ12, *B. subtilis* RJ46, and *Pseudomonas* sp. RJ15 (acidic agricultural soils); *Rhizobium* sp. PV-6 (*P. vulgaris* roots); *Sphingomonas* sp. Hbc-6; drought-recruited root-associated bacteria of alfalfa and *Arabidopsis*; *Massilia* spp.	ACC deaminase, antioxidant induction, recruitment of beneficial rhizosphere taxa	*Vigna mungo* and *Pisum sativum*; drought; contextual (Saikia et al., 2018) Red kidney bean (*P. vulgaris*); drought, transcriptome; contextual (Li et al., 2025a) Alfalfa; drought; contextual (Fan et al., 2023) Non-legume hosts; drought; contextual (Wang et al., 2022; Li et al., 2025b) Review (Amirhosseini et al., 2025)

ACC, 1-aminocyclopropane-1-carboxylate; EPS, exopolysaccharide(s); IAA, indole-3-acetic acid; PEG, polyethylene glycol; PGP, plant growth-promoting. Evidence category (per study): (A) extremophilic or extremotolerant strain evaluated in a legume under actual drought (soil water deficit; Section 2.1); (B) legume under salinity, heat, or PEG-induced osmotic stress; (C) non-legume host; (D) genomic, transcriptomic, biochemical, or *in vitro* evidence without *in planta* validation; NS, legume evaluated without stress (host compatibility only). Contextual: study meets host and stress criteria, but the strain does not meet the operational definition of extremophilic or extremotolerant (drought-adapted isolate from a non-extreme soil); not counted as direct evidence. Reviews are listed for context and are not classified.

## 2 Biological foundations: what makes a bioinoculant “extremophilic”

The application of extremophiles as bioinoculants is based on a premise that should be stated with precision: the adaptations that enable these microorganisms to survive in extreme environments could, in principle, translate into traits useful for sustaining plant growth under stress. However, this remains a working hypothesis rather than an established fact. Most of the mechanisms described in this section have been characterized at the bacterial level in culture, through genomic or transcriptomic analyses, or in *in vitro* assays and only a fraction has been evaluated in plants, with even fewer studies conducted in legumes under drought. This section therefore describes the functional repertoire of extremophiles as a mechanistic basis for their agronomic potential, while explicitly maintaining the distinction between documented capacity and demonstrated efficacy.

### 2.1 Operational definitions: extremophiles, extremotolerant bacteria, and drought

Extremophiles sensu stricto are organisms that require one or more extreme physicochemical conditions for optimal growth, whereas extremotolerant organisms grow optimally under moderate conditions but tolerate, and in some cases grow under, extreme conditions (Rothschild and Mancinelli, 2001). The distinction is based on growth rather than survival: survival of desiccation or of a transient stress does not qualify an organism as an extremophile. Commonly used criteria include optimal growth at 60–80 °C for thermophiles and above 80 °C for hyperthermophiles, optimal growth at pH < 3 for acidophiles and at pH > 9 for alkaliphiles, and a requirement for high salt concentrations for halophiles (2–5 M NaCl for extreme halophiles) (Rothschild and Mancinelli, 2001; Sahay et al., 2017; Arora and Panosyan, 2019). Xerophiles are defined by their capacity to grow at low water activity (a_w). Organisms able to complete their life cycle at a_w below approximately 0.8 are almost exclusively filamentous fungi, such as *Xeromyces bisporus* and some *Aspergillus* species, together with a few specialized halophilic archaea (Stevenson et al., 2015). Bacteria from dry soils and deserts are therefore better described as xerotolerant rather than xerophilic (Lebre et al., 2017). Polyextremophiles combine more than one of these adaptations, such as haloalkalithermophiles adapted to salinity, alkalinity, and high temperature (Mythrayee and Gayathri, 2024). Not all of these groups have the same relevance to drought tolerance. Xerotolerant microorganisms are the most directly pertinent, as their adaptations, including accumulation of compatible solutes, production of exopolysaccharides, biofilm formation, and metabolic reactivation after rehydration, respond to the same osmotic challenge imposed by water deficit (Lebre et al., 2017). Halotolerant and thermotolerant microorganisms are relevant in an adjacent sense: the osmotic component of salinity, together with the stability of their enzymes and membranes, shares part of its mechanistic basis with drought responses. Acidophiles and alkaliphiles have a more indirect relevance, mainly linked to nutrient availability in soils with extreme pH, and are therefore considered here in a more restricted manner.

Because most plant-associated isolates described as extremophilic in the agronomic literature have not been characterized against these growth criteria, this review uses three operational categories, maintained consistently throughout the text and in **Table 1** (**Figure 1A**): (i) extremophilic strains, for which optimal growth under an extreme condition has been experimentally documented; (ii) extremotolerant strains, which grow optimally under moderate conditions but were isolated from a documented extreme habitat (e.g., hot springs, hypersaline or hyperarid soils, Antarctic or high-altitude environments) or were shown to grow under extreme conditions; and (iii) drought-adapted or stress-tolerant isolates obtained from non-extreme agricultural or semi-arid soils and selected for tolerance *in vitro*, typically on PEG- or NaCl-amended media. Strains in category (iii) are functionally equivalent to conventional PGPB and are cited only as contextual evidence, identified as such. When the original study does not report growth optima, strains are designated as extremotolerant or by their habitat of origin rather than as extremophiles. Accordingly, extremophilic and extremotolerant strains are referred to collectively as extreme-environment-derived bacteria.

Drought is likewise defined operationally (**Figure 1B**). In this review, actual drought refers to a soil water deficit imposed on soil- or substrate-grown plants by withholding irrigation, deficit irrigation, or rain exclusion, and documented by at least one quantitative descriptor of soil water status, such as volumetric or gravimetric water content, percentage of field capacity or of available water, or soil matric potential. Studies are considered most informative when they also report plant water status (leaf water potential or relative water content), the duration and intensity of the deficit, and the developmental stage at which it was imposed. Withholding irrigation for a fixed number of days is not comparable across studies, because the resulting deficit depends on soil texture, pot volume, evaporative demand, and plant size. Osmotic stress imposed with polyethylene glycol (PEG) or mannitol in hydroponics, agar, or germination assays lowers water potential but does not reproduce soil drying, including changes in soil hydraulic conductivity, root–soil contact, nutrient diffusion, and microbial activity (Verslues et al., 2006). Such studies are therefore classified as adjacent or mechanistic evidence, not as evidence of drought tolerance.

**Table 1** summarizes, for each category of extremophilic or extremotolerant bacteria, representative strains, their documented functional traits, and the evidence supporting their application in plants, with the evidence category assigned to each individual study. Evidence is classified into four categories (**Figure 1C**): (A) direct evidence, corresponding to extremophilic or extremotolerant strains evaluated in legumes under actual drought, as defined above; (B) adjacent evidence in legumes, including studies conducted under salinity, heat, or PEG-induced osmotic stress, which are not considered evidence of drought tolerance; (C) evidence from non-legume hosts, where extremophilic or extremotolerant strains have been evaluated in other plant species; and (D) mechanistic evidence, comprising cellular, biochemical, genomic, transcriptomic, or *in vitro* studies without *in planta* validation. Studies conducted with conventional PGPB or with drought-adapted isolates from non-extreme soils, as well as studies performed without stress, are cited in the following sections only as contextual evidence and are identified as such. Genomic or *in vitro* evidence of a trait (category D) is interpreted as microbial capacity, not as evidence of trait expression in the rhizosphere or of plant protection under drought.

### 2.2 Genomic basis of stress tolerance

The ability of extremophilic and extremotolerant bacteria to sustain metabolic activity under drought-related osmotic, oxidative, and thermal stress relies on a coordinated set of genomic adaptations. These include molecular chaperones, Na^+^/H^+^ and other cation antiporters, compatible-solute biosynthesis and transport systems, antioxidant enzymes, exopolysaccharide (EPS) biosynthesis, and DNA repair mechanisms (Verma et al., 2018; Purohit et al., 2026). Moreover, bacterial genomes can be dynamically reprogrammed under stress by regulating genes involved in osmoregulation, oxidative stress defense, ion transport, and molecular repair, thereby optimizing survival under adverse environments (Igiehon et al., 2026).

The thermophilic strain *Bacillus paralicheniformis* CamBx3, isolated from a Chilean hot spring, provides a representative example of this adaptive repertoire. Its genome contains genes involved in EPS biosynthesis, assimilatory sulfate reduction, the heat-shock chaperones *dnaK* and *dnaJ*, and the Mrp Na^+^/H^+^ antiporter system, all of which contribute to stress adaptation (Narsing Rao et al., 2024). Functional analyses further showed that its EPS exhibits antioxidant and metal-chelating activities, traits potentially relevant to plant stress protection, although plant responses to water deficit were not evaluated in that study (Narsing Rao et al., 2024). Complementary transcriptomic evidence has also been reported. Under PEG-induced osmotic stress, the xerotolerant *Arthrobacter* sp. Helios exhibited extensive metabolic reprogramming in culture, including the induction of ABC transporters, compatible-solute uptake systems, and genes involved in iron homeostasis and antioxidant defense (Hernández-Fernández et al., 2022). Similar responses have been described across diverse extremophile groups, where molecular chaperones, ion transporters, osmoprotectant metabolism, and antioxidant systems constitute conserved adaptive mechanisms that support survival under multiple environmental stresses (Mesbah, 2022; Purohit et al., 2026). Collectively, these findings identify extreme-environment-derived bacteria as reservoirs of stress-adaptive genes. However, the presence of genes involved in EPS production, osmolyte metabolism, chaperones, ion transport, or antioxidant defense constitutes evidence of microbial capacity (category D); it does not demonstrate that these traits are expressed in the rhizosphere or that they protect the host plant under drought.

### 2.3 Plant–microbe signaling and extracellular microbial functions

Beyond intracellular gene regulation, rhizospheric and endophytic bacteria can interact with the host plant through chemical signals. Diverse biomolecules produced in the rhizosphere, including volatile organic compounds (VOCs), phytohormones, osmolytes, and siderophores, can be perceived by the plant and transduced through signaling pathways involving secondary messengers such as H_2_O_2_, Ca^2+^, and abscisic acid (ABA) (Sibanda et al., 2025). For example, *Bacillus subtilis* GB03 enhanced the synthesis of choline and of its derivative glycine betaine, a compatible solute, in *Arabidopsis thaliana*, and this response was associated with improved tolerance to osmotic stress and drought (Zhang et al., 2010). This example, however, involves a conventional PGPB rather than an extreme-environment-derived strain. For extremophiles, the available evidence in this context mostly describes microbial capacities rather than plant–microbe signaling. Halophilic and halotolerant bacteria isolated from crop rhizosphere soils produce haloenzymes, including cellulases, xylanases, proteases, amylases, and lipases that remain active under high salinity, together with compatible solutes (Reang et al., 2024). Similarly, extracellular enzymes produced by soil thermophiles can remain active for longer periods than those of mesophilic microorganisms, even under high temperatures and severe desiccation, contributing to soil nutrient cycling (Gonzalez et al., 2023). Neither study evaluated plant–microbe signaling or plant responses to water deficit; these findings therefore document microbial stress adaptation (category D) and should not be interpreted as evidence that these strains confer drought tolerance to legumes or other hosts.

### 2.4 Condition-dependent outcomes of inoculation

An often overlooked but critical aspect for the agricultural application of these bacteria is that the benefits of inoculation frequently depend on environmental conditions. For example, rhizobacteria with documented PGP potential improved root biomass in pepper (*Capsicum annuum*) only under drought, showing no beneficial effects under well-watered conditions (category C; Rolli et al., 2014). Similarly, among 74 halotolerant bacterial isolates, only three enhanced drought tolerance in durum wheat (*Triticum durum*), indicating that strains effective under salinity are not necessarily effective under water deficit (category C; Albdaiwi et al., 2019). These studies did not examine whether PGP traits were differentially expressed under stress. Condition-dependent benefits may therefore reflect changes in microbial gene expression, but also stress-induced changes in host physiology, such as altered root exudation, hormonal status, or carbon allocation, that modify the interaction independently of the bacterial phenotype. These findings highlight an important methodological consideration: the presence of PGP traits or positive *in vitro* results does not predict efficacy under drought. Microorganisms must be validated *in planta* and under the target stress before they can be considered reliable bioinoculants for drought-prone environments (Fadiji et al., 2022). The corollary that extreme-environment-derived bacteria may be particularly suited to water-limited conditions remains a hypothesis that requires testing under realistic agricultural settings, including comparisons under well-watered and water-deficit conditions within the same experiment. This principle underpins the framework adopted throughout this review, recognizing that a promising genomic or biochemical repertoire does not necessarily translate into agronomic efficacy.

## 3 Physiological mechanisms induced by extremophilic bacteria in legumes under drought

Building on the framework outlined above, this section examines how extremophilic and extremotolerant bacteria may translate their adaptive repertoire into physiological changes in legume hosts, encompassing rhizospheric water retention, root system remodeling, and the hormonal control of nodule formation and function under drought. For each mechanism, evidence obtained with conventional PGPB, which is more abundant, is distinguished from evidence obtained with extreme-environment-derived strains.

### 3.1 Exopolysaccharides, biofilms, and rhizospheric water retention

Several bacteria isolated from saline and thermal environments produce exopolysaccharides and form biofilms. These extracellular polymers, composed mainly of sugars such as glucose, mannose, galactose, fructose, and raffinose, together with proteins and nucleic acids, form a highly hydrated matrix (Sen et al., 2025). In soil, biofilm formation can stabilize aggregates, improve soil structure, and enhance water retention in the rhizosphere, creating a hydrated microenvironment that may reduce water loss and facilitate water uptake during drought episodes (Ghosh et al., 2019; Zhakypbek et al., 2026). Genome analysis of the thermophilic strain *B. paralicheniformis* CamBx3 revealed EPS biosynthetic clusters and stress-response genes (Narsing Rao et al., 2024); this indicates a capacity for EPS production (category D) but does not establish a protective role of this EPS *in planta*.

EPS production has been documented in strains of *Pseudomonas* and *Bacillus*, among other genera, and has been associated with protection of bacterial cells, and potentially of roots, against desiccation, salinity, and high temperatures (Banerjee et al., 2021; Kumari et al., 2024); however, EPS yield and composition vary widely among strains within these genera. Under stress conditions, EPS production and composition are dynamically modified, increasing the accumulation of carbohydrates, proteins, and compatible sugars that function as osmoprotectants and carbon reservoirs (Igiehon et al., 2025). This hydrated polysaccharide matrix may enhance water availability in the rhizosphere, improve soil aggregation, and bind toxic ions, particularly Na^+^, thereby contributing to ion homeostasis under salinity (Ghosh et al., 2019). These functions have been reported in different plant–microbe systems, mostly involving conventional PGPB. EPS-producing strains of *Pseudomonas* sp. AK-1, *Bradyrhizobium japonicum*, and *B. subtilis* restricted Na^+^ uptake by soybean roots under salinity stress (Morcillo and Manzanera, 2021). Similarly, the conventional PGPB *Azospirillum brasilense* Sp245 synthesized high-molecular-weight carbohydrate complexes under desiccation. These polymers increased water availability in the rhizosphere and extended the time available for plant metabolic adjustment to drought (Morcillo and Manzanera, 2021).

For extreme-environment-derived strains, halotolerant *Bacillus* strains isolated from the Atacama Desert improved water status and nutrient uptake in drought-stressed lettuce (category C) (Santander et al., 2024). In legumes, halophilic rhizobacteria producing EPS and biofilms enhanced soil aggregation and promoted chickpea (*Cicer arietinum*) growth under salinity stress (category B) (Qurashi and Sabri, 2012). Together, these studies support EPS formation as a plausible mechanism for improving plant water status, but its contribution in legumes under actual drought has not yet been validated with extreme-environment-derived strains (no category A evidence) (**Figure 2A**).

**Figure 2:**
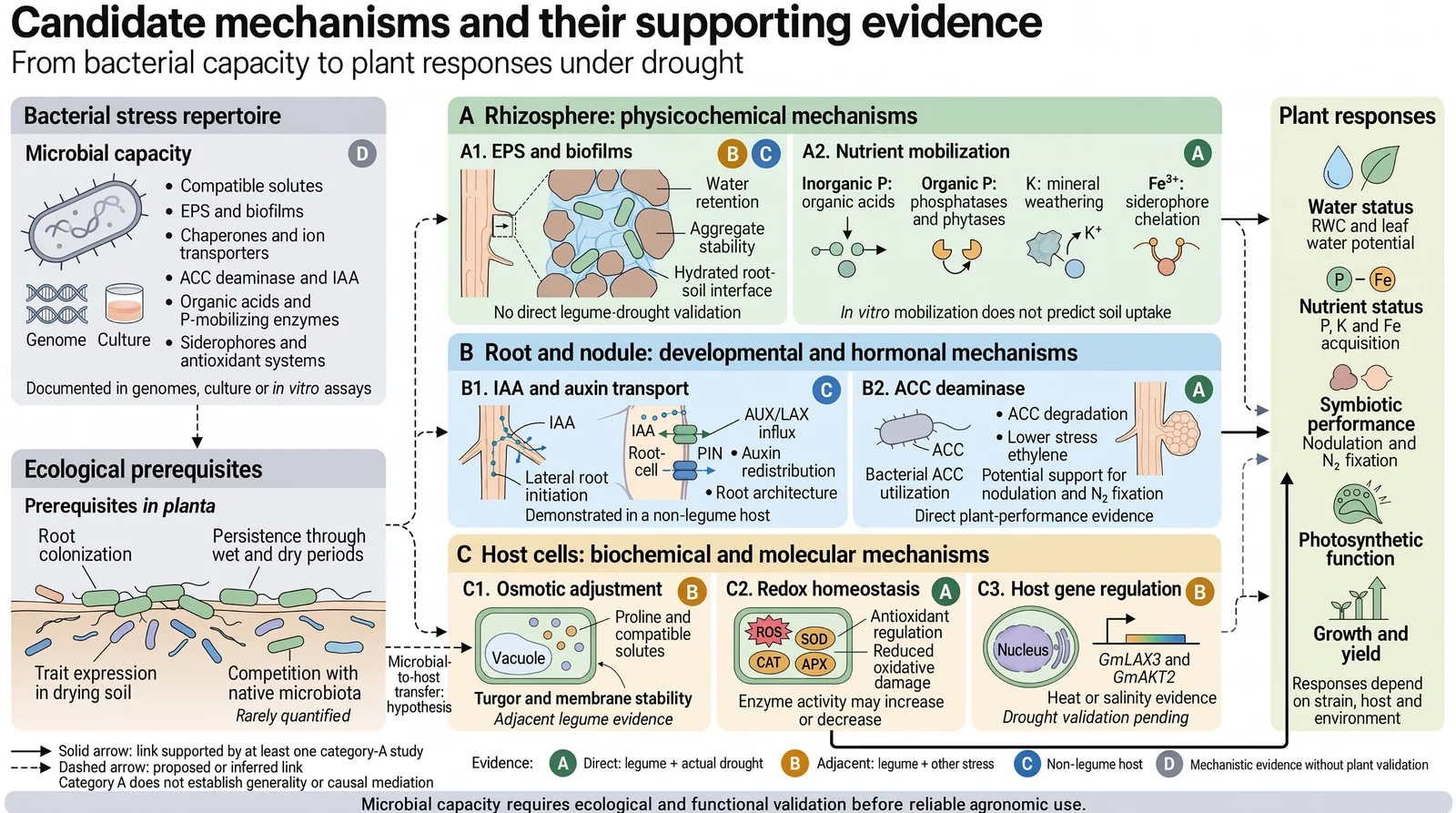
Mechanisms through which extremophilic and extremotolerant bacteria may contribute to drought tolerance in legumes, organized by level of organization. The bacterial stress repertoire (left) represents microbial capacity documented in genomes, culture, or *in vitro* assays (category D); its translation into plant effects requires root colonization, persistence, and trait expression in drying soil, which are rarely quantified. **(A)** Rhizosphere, physicochemical mechanisms: EPS and biofilm-mediated water retention and aggregate stability (A1), and nutrient mobilization (A2), in which inorganic P is solubilized mainly by organic acids and organic P is mineralized by phosphatases and phytases, K is released by mineral weathering, and Fe^3+^ is chelated by siderophores. **(B)** Root and nodule, developmental and hormonal mechanisms: IAA-dependent modulation of AUX/LAX and PIN-mediated auxin transport (B1), and ACC deaminase-mediated reduction of stress ethylene supporting nodulation and N_2_ fixation (B2). **(C)** Host cells, biochemical and molecular mechanisms: osmotic adjustment (C1), redox homeostasis (C2), and regulation of host stress-responsive genes (C3). Colored symbols indicate the highest evidence category available for extremophilic or extremotolerant strains for each mechanism [**(A–D)** as defined in **Figure 1** and **Table 1**]; category A indicates at least one study and does not imply that the mechanism is generally established. Solid arrows indicate links supported by at least one category-A study; dashed arrows indicate links proposed or inferred from adjacent, non-legume, or mechanistic evidence.

### 3.2 Root architecture and auxin homeostasis

Bacteria can also influence root system architecture by modulating the homeostasis of phytohormones, particularly auxins. Many rhizobacteria, including strains isolated from extreme environments, produce indole-3-acetic acid (IAA). Rather than acting only as an exogenous hormone source, microbially derived IAA can modulate polar auxin transport in roots through AUX/LAX influx and PIN efflux carriers (Defez et al., 2017; Marasco et al., 2024). For example, the desert-derived strains *Pseudomonas* spp. E102 and E141, isolated from date palm roots, colonized roots, increased auxin levels, inhibited primary root growth, and promoted root hair elongation and lateral root formation; these effects depended on the host auxin response and transport machinery and were attenuated in IAA-deficient mutant strains, indicating that the bacteria act through, rather than replace, the endogenous auxin system (Marasco et al., 2024). These effects were described in *Arabidopsis thaliana* under salinity (category C) and have not been evaluated in legumes under drought. In general terms, a modified root architecture may improve soil exploration and water and nutrient uptake under drought, stimulate root exudation, and promote interactions with other beneficial microorganisms (Mikiciuk et al., 2024).

Evidence from legumes under water deficit derives mainly from conventional or genetically modified rhizobia and PGPB. In alfalfa (*Medicago sativa*), *Rhizobium* strains engineered to overproduce IAA enhanced drought tolerance through interactions with endogenous ABA, jasmonate, and ethylene signaling pathways (Defez et al., 2017). Similarly, inoculation of *P. vulgaris* with the conventional PGPB *Azospirillum brasilense* Cd increased root length and root projection area, producing longer and thinner roots, and increased taproot length under water stress in an inoculum-dependent manner, without increasing plant biomass (German et al., 2000; reviewed by Chieb and Gachomo, 2023). Together, these studies indicate that bacterial modulation of auxin signaling can contribute to root development and drought adaptation in legumes; however, this mechanism has not yet been demonstrated for an extremophilic or extremotolerant strain in a legume under actual drought (**Figure 2B**).

### 3.3 Nodulation, ACC deaminase, and auxin/ethylene balance

Hormonal modulation by rhizobacteria also plays a key role in nodule formation and function in legumes, a process regulated by the balance between auxin and ethylene (Abd-Alla et al., 2024). Auxins promote cortical cell division, nodule organogenesis, and delay nodule senescence. However, excessive auxin levels stimulate 1-aminocyclopropane-1-carboxylate synthase (ACS), increasing ethylene production, which accelerates nodule senescence and suppresses biological nitrogen fixation (Gamalero and Glick, 2022). To counteract this effect, bacteria expressing 1-aminocyclopropane-1-carboxylate deaminase (ACC deaminase), including isolates from saline and arid environments, degrade ACC, the immediate precursor of ethylene, thereby lowering ethylene levels while preserving the beneficial effects of IAA on plant growth and nodulation (Saikia et al., 2018; Reang et al., 2024; Sathya et al., 2024).

Because nodulation is a defining feature of legumes and one of the first processes impaired by drought, maintaining nodule function is essential for sustaining biological nitrogen fixation. Plant growth-promoting rhizobacteria can help preserve nodule initiation, delay nodule senescence, and maintain N_2_ fixation under drought and salinity stress (Alemneh et al., 2020). Evidence for extreme-environment-derived strains is more limited. Inoculation of soybean with the Antarctic strain *Pseudomonas* sp. ATCC PTA-122608 increased nodulation by more than 100% compared with non-inoculated plants (Garcia et al., 2021); however, this experiment was conducted without water stress and therefore indicates symbiotic compatibility rather than a contribution to drought tolerance. In *Vigna mungo* and *Pisum sativum*, an ACC deaminase-producing bacterial consortium isolated from acidic soils reduced ACC accumulation and improved plant performance under drought (Saikia et al., 2018); because the isolation habitat does not meet the criteria defined in Section 2.1, these strains are considered stress-tolerant isolates rather than extremophiles. Direct evidence for extremotolerant strains in legumes under drought is available for chickpea, in which about half of ten stress-tolerant strains associated with chasmophytic wild *Chenopodium* produced ACC deaminase and several of them improved growth, nutrient content, and field yield under water stress (category A) (Das et al., 2024), and for clusterbean, in which multi-trait isolates from arid soils of western Rajasthan, including *B. badius* G8-6, *B. paralicheniformis* G11-5, and *Stenotrophomonas pavanii* G12-4, increased plant dry weight, plant water potential, and leaf relative water content in drought-stressed pots and yield under rainfed conditions (category A) (Kasana et al., 2023). In neither study was the contribution of ACC deaminase separated from that of other PGP traits. The most complete mechanistic evidence, however, comes from a conventional rhizobacterium: *Variovorax paradoxus* 5C-2 attenuated the drought-induced increase in xylem ACC and prevented drought-induced decreases in nodulation and seed nitrogen content in *P. sativum* grown in drying soil, whereas a mutant with strongly reduced ACC deaminase activity did not improve plant growth, providing causal evidence for the role of this enzyme (Belimov et al., 2009) (**Figure 2B**).

## 4 Biochemical mechanisms induced by extremophilic bacteria in legumes under drought

In addition to these physiological adjustments, bacterial inoculation can modulate the biochemical status of the host plant. The following section addresses the contribution of extremophilic, extremotolerant, and conventional strains to osmotic adjustment through compatible-solute accumulation and to the reinforcement of antioxidant defenses against drought-induced oxidative stress.

### 4.1 Compatible osmolytes and osmotic adjustment

Under drought, the decline in soil water potential imposes osmotic stress, leading to loss of cell turgor, membrane destabilization, and protein denaturation. Bacteria adapted to saline and dry environments synthesize or accumulate compatible osmolytes such as trehalose, proline, glycine betaine, sucrose, and glutamate, which contribute to their own osmotic adjustment while stabilizing proteins and cellular membranes (Kumar et al., 2024). It has been proposed that microbial osmolytes released in the rhizosphere may be taken up by roots and reduce the metabolic cost of *de novo* osmolyte synthesis by plants; this remains a hypothesis, because the transfer of bacterial osmolytes to host tissues has rarely been quantified. In addition, microbial communities adapted to chronically arid environments exhibit greater resilience to recurrent drought (Moore et al., 2023), although the consequences of this microbial legacy for plant performance require direct evaluation.

Evidence from legumes supports the importance of compatible osmolytes in bacteria-mediated drought tolerance, although it derives largely from conventional or genetically modified strains. In *P. vulgaris*, inoculation with a *Rhizobium etli* strain overexpressing trehalose-6-phosphate synthase enhanced the expression of genes involved in carbon and nitrogen metabolism, improved plant recovery, and increased grain yield by more than 50% under drought (Suárez et al., 2008). Similar responses have been reported in *A. hypogaea*, where osmolyte-producing bacterial inoculants increased the accumulation of proline, trehalose, glycine betaine, and salicylic acid, leading to improvements in plant growth, physiological performance, and yield under water deficit (Eswaran et al., 2024). Likewise, inoculation with a consortium of *R. pusense* S6R2 and *Enterobacter cloacae* S23 enhanced osmolyte accumulation together with nodulation, photosynthetic pigments, antioxidant activity, and plant growth under drought conditions (Ramakrishnan et al., 2024). Collectively, these findings identify osmotic adjustment as one of the best-supported biochemical mechanisms through which bacterial inoculants enhance drought tolerance in legumes. However, an extreme-environment origin was not reported for these strains, and the relative contribution of bacterial versus host-synthesized osmolytes was not resolved. For extreme-environment-derived strains, the evidence is indirect: halotolerant strains increased proline accumulation in soybean under salinity (category B) (Yasmin et al., 2020), whereas under drought, inoculated clusterbean accumulated less proline than non-inoculated plants (Kasana et al., 2023), which is consistent with reduced stress rather than with enhanced osmotic adjustment (**Figure 2C**).

### 4.2 Redox homeostasis and antioxidant systems

Drought disrupts cellular redox homeostasis by promoting the accumulation of reactive oxygen species (ROS), leading to oxidative damage of lipids, proteins, and membranes, together with impaired photosynthetic performance. Bacterial inoculation can alleviate these effects through complementary antioxidant strategies. Inoculated plants frequently show enhanced activities of superoxide dismutase (SOD), catalase (CAT), and ascorbate peroxidase (APX), while bacterial exopolysaccharides (EPS) may provide additional protection by scavenging free radicals and contributing to osmotic adjustment and ionic homeostasis (Ravi et al., 2023; Srivastava et al., 2024). Their lipid and polysaccharide components exhibit emulsifying and free radical-scavenging properties that help alleviate ROS-induced cellular damage (Sarkar et al., 2024). Halotolerant strains such as *B. subtilis* NRC1aza and *B. cereus* AG3 produce EPS with strong antioxidant activity, whereas thermophilic strains including *B. anthracis* PFAB2 and *B. haynesii* CamB6 synthesize thermostable EPS with high water-holding capacity, antioxidant activity, and emulsifying properties (Díaz-Cornejo et al., 2023; Kumari et al., 2024). Because *B. anthracis* is a regulated pathogen, the relevance of strain PFAB2 is restricted to EPS characterization. Together, these *in vitro* characteristics suggest that EPS from extreme-environment-derived strains could contribute to both water retention and ROS scavenging, a hypothesis that has not yet been tested in legumes under drought (category D).

Evidence from legumes indicates that bacterial inoculation can reinforce plant antioxidant defenses under drought, although most of the strains evaluated are not of documented extreme origin. The ACC deaminase-producing consortium *Ochrobactrum pseudogrignonense* RJ12, *B. subtilis* RJ46, and *Pseudomonas* sp. RJ15, isolated from acidic soils, increased chlorophyll, proline, phenolic compounds, and the activities of antioxidant enzymes, including SOD, CAT, and POD, in *V. mungo* and *P. sativum* under drought (Saikia et al., 2018). Similar responses have been reported in *C. arietinum*, *A. hypogaea*, and *V. faba*, where inoculation with *Mesorhizobium* spp., *Acinetobacter calcoaceticus* AC06, *B. amyloliquefaciens* BA01, and the drought-tolerant rhizobial strain QHCD11 enhanced SOD, CAT, and APX activities, reduced oxidative damage, and improved plant growth and yield under drought conditions (Khan et al., 2019; Eswaran et al., 2024; Li et al., 2023). Among these, strain QHCD11, isolated from faba bean on the Qinghai–Tibet Plateau, is the only one whose high-altitude origin could qualify it as extremotolerant (category A) (Li et al., 2023). In contrast, stress-tolerant strains associated with chasmophytic plants reduced antioxidant enzyme activities in water-stressed chickpea (category A) (Das et al., 2024), and arid-soil isolates reduced antioxidant enzyme activities and proline in drought-stressed clusterbean (category A) (Kasana et al., 2023), which is consistent with alleviation of oxidative stress rather than enhanced ROS scavenging. Depending on the physiological status of the plant, inoculation may therefore either stimulate antioxidant enzyme activity or lower it by reducing ROS production. Both responses may reflect improved redox homeostasis, and enzyme activities alone cannot discriminate between them without concurrent measurements of oxidative damage and plant water status.

Complementary evidence from heat and salinity stress (category B) indicates that antioxidant responses are shared across abiotic stresses. In soybean, the thermotolerant strain *B. cereus* SA1 enhanced chlorophyll, carotenoid, SOD, APX, and glutathione (GSH) contents while reducing lipid peroxidation and improving photosystem II efficiency under heat stress (Khan et al., 2020b), with comparable thermoprotective effects previously reported in tomato seedlings inoculated with the same isolate (category C) (Khan et al., 2020a). Likewise, halotolerant *P. pseudoalcaligenes* and *B. subtilis* improved photosynthetic performance, increased proline accumulation, and reduced malondialdehyde levels in soybean under salinity (Yasmin et al., 2020). In groundnut, strains described as extremophilic, *P. extremorientalis*, *Olivibacter soli*, and *Pseudomonas poae*, increased chlorophyll, carotenoid, and total phenolic contents under saline conditions (Tanur Erkoyuncu et al., 2026). These findings indicate that antioxidant responses are not specific to drought, but they do not constitute evidence of drought tolerance, and direct validation under drought in legumes remains limited (**Figure 2C**).

## 5 Mechanisms of extremophile-mediated nutrient acquisition underlying drought tolerance in legumes

Beyond these physiological and biochemical effects, bacterial inoculants may contribute to legume drought tolerance by improving the acquisition of phosphorus, potassium, and iron, three nutrients whose availability declines markedly under water deficit and whose supply is closely coupled to photosynthetic performance, osmotic regulation, and biological nitrogen fixation.

### 5.1 Phosphorus solubilisation and coupling to N_2_ fixation

Phosphorus (P) availability declines markedly under drought because reduced soil moisture limits phosphate diffusion toward the root surface. This constraint is particularly critical in legumes, where biological nitrogen fixation (BNF) is a highly energy-demanding process that relies on adequate phosphorus supply for ATP production, nitrogenase activity, and nodule development (Fadiji et al., 2022; Poudel et al., 2021; Rajasreelatha and Thippeswamy, 2022). Phosphate-solubilizing bacteria, including some extremotolerant isolates, may partially alleviate this limitation through two complementary processes. Inorganic P is mobilized from sparingly soluble Ca-, Fe-, and Al-phosphates mainly by the release of organic acids, such as gluconic, citric, lactic, and succinic acids, which lower rhizosphere pH and chelate the cations bound to phosphate, whereas organic P is mineralized by phosphatases, phytases, and phosphonatases acting on phosphomonoesters, phytate, and phosphonates, respectively (Richardson and Simpson, 2011; Benmrid et al., 2023). Among these mechanisms, oxidation of glucose to gluconic acid through the pyrroloquinoline quinone (PQQ)-dependent glucose dehydrogenase (GDH) pathway is considered one of the most efficient systems for solubilizing inorganic phosphate (Alori et al., 2017; Joshi et al., 2023). Consistent with this mechanism, the halotolerant bacterium *Bacillus tequilensis*, carrying the *pqqE* gene, enhanced phosphorus solubilization and improved relative water content, photosynthetic pigments, and antioxidant activity in chickpea under saline conditions (category B) (Haroon et al., 2023).

Phosphate solubilization measured *in vitro* should nevertheless be interpreted with caution. Plate and liquid assays commonly use tricalcium phosphate, which is readily dissolved by modest acidification and is a weak predictor of P mobilization in most soils (Bashan et al., 2013). In drying soil, P release and uptake further depend on soil chemistry, water content, phosphate diffusion toward roots, root activity, and the survival and metabolic activity of the inoculant, all of which decline as soil water potential decreases. Consequently, *in vitro* solubilization capacity (category D) does not by itself indicate improved P nutrition under drought; the same caution applies to potassium solubilization (Section 5.2).

Direct evidence under drought has been reported in chickpea, where stress-tolerant bacteria isolated from chasmophytic wild *Chenopodium*, including *B. paralicheniformis* L38, *Pseudomonas* sp. LN75, *Enterobacter hormaechei* subsp. *xiangfangensis* LJ89, *B. paramycoides* L17, and *Micrococcus luteus* LA9, solubilized phosphorus, potassium, and zinc *in vitro*, increased N, P, K, Fe, and Zn contents in chickpea under water stress in the greenhouse, and improved grain yield under water stress in the field (category A) (Das et al., 2024). Other studies in common bean, mungbean, chickpea, faba bean, and garden pea have shown that phosphate-solubilizing bacteria, including *Bradyrhizobium* spp., *Agrobacterium* sp., *Bacillus aryabhattai*, *Bacillus subtilis*, *Bacillus flexus*, *Pseudomonas* spp., *Enterobacter* spp., and *Azospirillum* sp., increased phosphorus uptake, nodulation, nitrogen accumulation, plant growth, and grain yield under phosphorus deficiency or salinity (Zafar et al., 2011; Ahmad et al., 2019; Midekssa et al., 2016; Janati et al., 2023; Kumawat et al., 2021; 2023). These studies involved conventional PGPB and were not conducted under drought; they support the coupling between P acquisition and BNF but do not constitute evidence of extremophile-mediated drought tolerance. Collectively, improved phosphorus acquisition appears closely linked to BNF in legumes, whereas its contribution to drought tolerance conferred by extreme-environment-derived strains rests on a small number of studies (Das et al., 2024; Kasana et al., 2023).

### 5.2 Potassium solubilisation and osmotic homeostasis

Potassium (K) plays a central role in drought tolerance by regulating stomatal movement, osmotic adjustment, enzyme activation, and cellular turgor, thereby sustaining photosynthesis and water-use efficiency under water deficit (Etesami et al., 2017). Some bacteria, including stress-tolerant isolates, can increase K availability by weathering potassium-bearing minerals through the secretion of organic acids and other metabolites that release K^+^ into the rhizosphere (Olaniyan et al., 2022). In addition, bacterial auxin production promotes root development and soil exploration, which may further increase potassium acquisition by plants (Upadhyay et al., 2022). As for P, *in vitro* K solubilization does not necessarily translate into improved K nutrition in drying soil, where K^+^ diffusion toward roots is strongly restricted.

Direct evidence under drought comes from the chickpea study described above, in which K-solubilizing strains, including *Bacillus paralicheniformis* L38, *Pseudomonas* sp. LN75, and *Enterobacter hormaechei* subsp. *xiangfangensis* LJ89, increased plant K content under water stress in the greenhouse and were identified as the most promising inoculants on the basis of field yield under water stress (category A) (Das et al., 2024).

Complementary evidence from other legumes derives from conventional potassium-solubilizing bacteria evaluated without water stress. In chickpea, potassium-solubilizing isolates increased K uptake and biomass production under different soil K levels (Patel et al., 2024). Under salinity, salt-tolerant *Rhizobium* and ACC deaminase-producing rhizobacteria improved potassium nutrition, symbiotic performance, antioxidant activity, and grain yield in mungbean and chickpea (category B) (Kumawat et al., 2021; 2023). These results support a role of K acquisition in osmotic homeostasis under salinity, but its contribution under drought for extreme-environment-derived strains remains to be established.

### 5.3 Siderophores and iron acquisition

Iron (Fe) is essential for photosynthesis, respiration, antioxidant metabolism, and biological nitrogen fixation, but its availability declines under drought because Fe is rapidly oxidized into poorly soluble forms in dry, aerobic soils (Al-Turki et al., 2023; Fadiji et al., 2022). Many bacteria, including extreme-environment-derived strains, produce siderophores, low-molecular-weight compounds that chelate Fe^3+^ and increase its availability for both plants and microorganisms. Improved iron acquisition may support chlorophyll biosynthesis, antioxidant enzyme activity, and nodule function, linking Fe nutrition with redox homeostasis and symbiotic nitrogen fixation (Timofeeva et al., 2022).

Evidence under drought in legumes remains limited. In the chickpea study described above, the chasmophyte-associated strains increased plant Fe and Zn contents under water stress in the greenhouse (category A) (Das et al., 2024). In soybean, siderophore-producing bacteria were associated with improved seed germination, iron homeostasis, and nodulation under water deficit or combined Fe deficiency and water stress (Igiehon et al., 2019; Peter et al., 2024; Hasan et al., 2025). However, the germination assays of Igiehon et al. (2019) were conducted under PEG-induced osmotic stress (category B), and an extreme-environment origin was not reported for the strains evaluated in these soybean studies.

Complementary evidence from salinity and other crop species is consistent with this mechanism. Siderophore-producing bacteria increased chlorophyll content, antioxidant activity, micronutrient uptake, and plant growth under saline conditions, while several reviews highlight their dual contribution to plant stress tolerance by improving Fe nutrition and limiting pathogen development through competition for iron (Singh et al., 2022b; Ahmad et al., 2022). Together, these findings identify siderophore-mediated iron acquisition as a plausible nutritional mechanism connecting micronutrient acquisition, antioxidant protection, and symbiotic performance under abiotic stress, although further validation under drought in legumes is still required (**Figure 2A**).

## 6 Extremophile-mediated gene regulation in legumes

Bacteria-mediated drought tolerance in legumes involves molecular responses in both the bacterial partner and the host plant. In stress-tolerant rhizobia, osmotic stress does not suppress symbiotic signaling but instead reconfigures its regulatory network, allowing continued expression of nodulation genes such as *nodD* and *nodABC* and sustained production of Nod factors required for host recognition, infection-thread formation, and biological nitrogen fixation (del Cerro et al., 2019; Fuentes-Romero et al., 2023; Tiwari et al., 2024; Ben Gaied et al., 2024). These data were obtained mainly under saline or osmotic conditions and therefore represent adjacent or mechanistic evidence. In parallel, auxin- and ACC deaminase-producing bacteria indirectly regulate ethylene-related gene expression, reducing ethylene accumulation, delaying nodule senescence, modulating root architecture, and helping to maintain nitrogen fixation under drought and salinity (Saikia et al., 2018; Alemneh et al., 2020).

Bacterial inoculation can also modulate host hormone signaling. In soybean under drought, the combined application of melatonin and *Bacillus* sp. IPR-4 upregulated genes involved in ABA biosynthesis and signaling, including *GmNCED3*, *GmDREB2*, and *GmbZIP1*, together with the phenylpropanoid-pathway gene *GmPAL2.1* (Peter et al., 2024). Because this response was associated with a combined treatment and an extreme-environment origin was not reported for this strain, it cannot be attributed to extremophile-specific traits. Beyond hormonal regulation, some studies indicate that extreme-environment-derived bacteria modulate host genes involved in root development, ion transport, and antioxidant defense, although under stresses other than drought. In soybean, the thermotolerant *Bacillus cereus* SA1 restored the expression of the auxin transporter *GmLAX3* and the potassium channel *GmAKT2* under heat stress (category B) (Khan et al., 2020b). Likewise, halophilic strains of *Stenotrophomonas* and *Exiguobacterium* induced genes associated with plant growth, water homeostasis, and stress protection in soybean under salinity (category B) (Santos et al., 2023). More generally, transcriptomic analyses of abiotic stress responses identify members of the DREB, NAC, WRKY, and bZIP transcription factor families as central regulators coordinating antioxidant defenses, osmotic adjustment, and hormone signaling (Feng et al., 2023; Sibanda et al., 2025; Hou et al., 2024; Igiehon et al., 2026; Li et al., 2019); whether extremophilic or extremotolerant inoculants act through these networks in legumes under drought has not been tested (**Figure 2C**). Collectively, these studies suggest that extreme-environment-derived bacteria can modulate host gene networks associated with symbiosis, hormone signaling, ion homeostasis, and stress responses. The closest approach is the study of Li et al. (2025a), in which *Rhizobium* sp. PV-6, isolated from *P. vulgaris* roots, improved growth, root traits, and nodulation of red kidney bean under drought across seven growth stages and modulated host stress-related gene expression and root-associated microbial communities; this strain, however, is not of extreme-environment origin. Therefore, no study has yet combined a documented extreme-origin inoculant, a legume host, actual drought, and host transcriptomic analysis (**Figure 3**, G1).

**Figure 3:**
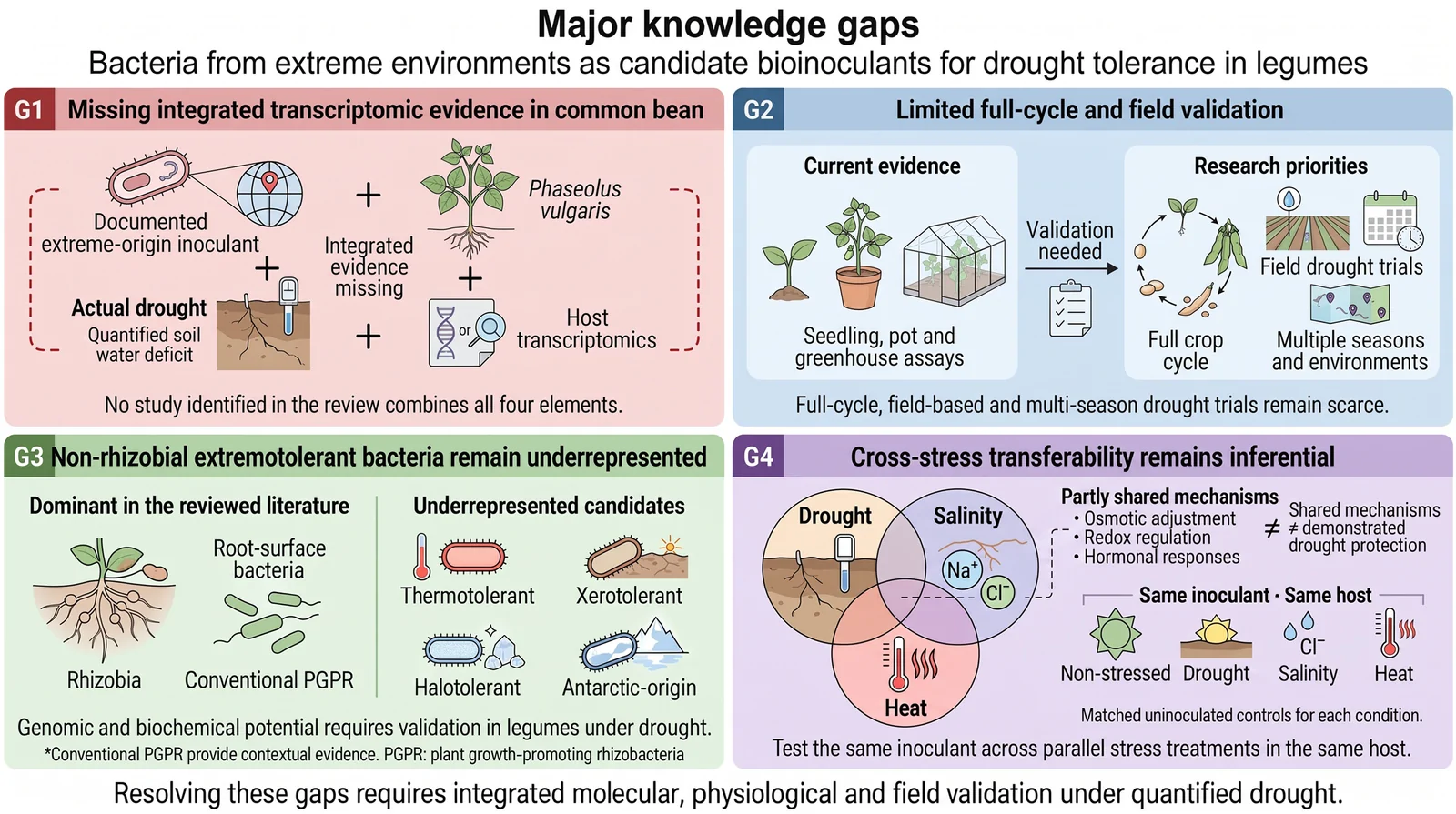
Major knowledge gaps limiting the development and validation of bioinoculants based on extremophilic and extremotolerant bacteria for improving drought tolerance in legumes. (G1) highlights the absence of studies that simultaneously integrate a documented extreme-origin inoculant, *P. vulgaris*, actual drought, and host transcriptomic analysis. (G2) emphasises the scarcity of full-phenological-cycle, multi-season, and field-based drought trials, as most direct evidence remains concentrated in seedling, pot, or greenhouse assays. (G3) shows that direct evidence is still dominated by rhizobia and conventional plant growth-promoting rhizobacteria, whereas non-rhizobial extremotolerant bacteria, including thermotolerant, xerotolerant, halotolerant, and Antarctic-origin strains, remain underrepresented despite their genomic and biochemical potential. (G4) illustrates the frequent use of salinity and heat as substitutes for drought; although these stresses share partially overlapping mechanisms, cross-stress transferability remains inferential when identical inoculants are not tested across parallel stress treatments in the same host.

## 7 Limitations and ecological constraints of extreme-environment-derived inoculants

The traits that allow bacteria to persist in extreme habitats do not necessarily confer rhizosphere competence. Root colonization requires chemotaxis toward root exudates, motility, attachment and biofilm formation on root surfaces, efficient use of exudate-derived carbon, and compatibility with plant immune responses. Strains isolated from habitats without plants, such as hot springs, hypersaline lakes, or bare desert soils, may lack these traits. Moreover, obligate extremophiles, whose growth optimum lies outside the temperature, pH, or salinity range of agricultural soils, are unlikely to grow, or even to remain metabolically active, in the rhizosphere of a legume crop. Consistent with this constraint, most strains with reported plant benefits in **Table 1** are extremotolerant rather than extremophilic, and several were isolated from plant-associated niches within extreme environments, such as desert plants or chasmophytes (Marasco et al., 2024; Das et al., 2024). Root colonization and inoculant persistence were rarely quantified in the studies reviewed here, which prevents distinguishing effects of an established inoculant from transient effects of the inoculum or its metabolites.

A second constraint concerns performance under non-extreme conditions. Adaptations to extreme environments, such as the synthesis of compatible solutes, EPS, and stress proteins, carry metabolic costs that may reduce growth rate and competitiveness when stress is relaxed. Under moderate soil water availability, extreme-environment-derived inoculants may therefore be outcompeted by native microorganisms better adapted to local conditions, and their effect on the plant may be neutral, as reported for drought-dependent effects of PGPB (Rolli et al., 2014). Because drought in rainfed legume production is usually intermittent, an inoculant must persist through well-watered periods to be present when water deficit develops. Introduced strains commonly decline after inoculation because of biotic resistance of resident communities and unfavorable soil conditions, and inoculation can also modify resident microbial communities in ways that are not always reversible (Kaminsky et al., 2019; Mawarda et al., 2020; O’Callaghan et al., 2022).

A third constraint is biosafety. Several genera frequently reported among stress-tolerant PGPB, including *Stenotrophomonas*, *Acinetobacter*, *Enterobacter*, *Ochrobactrum*, and the *B. cereus* group, contain opportunistic human pathogens (Berg et al., 2005), and one strain cited in this review for its EPS properties belongs to *B. anthracis*. Strain-level, genome-based safety assessment, including screening for virulence factors and antibiotic-resistance genes, should therefore precede field release, and taxonomic assignment at the genus level is insufficient for this purpose.

These constraints suggest several strategies. First, candidate strains may be prioritized among extremotolerant bacteria isolated from plant-associated niches in extreme environments, such as the rhizosphere, endosphere, or nodules of desert, halophytic, or high-altitude legumes, which combine stress tolerance with host-association traits. Second, rhizosphere competence should be incorporated into screening pipelines by quantifying root colonization and persistence in non-sterile soil with strain-specific qPCR, marker genes, or strain-resolved sequencing, rather than selecting strains on the basis of *in vitro* PGP traits alone. Third, candidate strains should be evaluated under both well-watered and water-deficit conditions within the same experiment, to determine whether benefits are condition-dependent and whether inoculation carries a penalty in the absence of stress. Fourth, consortia may combine stress-tolerant strains with locally adapted, rhizosphere-competent strains or compatible rhizobia, so that establishment and stress-related functions are distributed among members. Fifth, formulations and delivery methods that protect viability and favor early establishment, such as seed coating, encapsulation, or carrier-based inoculants, together with repeated application at critical phenological stages, may improve persistence (Bashan et al., 2014; O’Callaghan et al., 2022). Finally, effects on resident microbial communities should be monitored, in line with the non-target effects documented for other inoculants (Mawarda et al., 2020).

## 8 Knowledge gaps and research hypotheses

Despite the growing body of literature on extremophilic bacteria and plant stress tolerance, important knowledge gaps continue to limit the translation of current findings into agricultural applications. These limitations are not restricted to the number of available studies but also involve the type and strength of the experimental evidence. Direct evaluations of extremophilic or extremotolerant bacteria in legumes under actual drought conditions remain scarce, whereas much of the current knowledge is derived from related abiotic stresses, non-legume hosts, or mechanistic studies without agronomic validation. In addition, non-rhizobial extremotolerant bacteria remain largely unexplored, and most experiments have been conducted under controlled conditions or during early developmental stages, with relatively few studies assessing the complete crop cycle or validating inoculants under field conditions. Collectively, these limitations identify key priorities for future research (**Figure 3**).

Based on these knowledge gaps, three major hypotheses emerge. (H1) Adaptive mechanisms that enable extremophilic and extremotolerant bacteria to survive under salinity, heat, or other extreme conditions may also confer drought tolerance in legumes, although this transferability remains to be demonstrated under actual water-deficit conditions. (H2) Non-rhizobial extremotolerant bacteria may represent an underexplored source of bioinoculants capable of complementing conventional plant growth-promoting bacteria, particularly under severe or fluctuating drought conditions, provided that they establish and persist in the rhizosphere. (H3) Integrating physiological, biochemical, molecular, and agronomic approaches throughout the complete crop cycle and under field conditions will provide the evidence required to identify the mechanisms that are consistently translated into improved plant performance and yield in legumes.

## 9 Concluding remarks and future directions

Current evidence indicates that extremophilic and, more commonly, extremotolerant bacteria possess traits that could contribute to drought tolerance in legumes, including EPS production, ACC deaminase activity, osmolyte synthesis, antioxidant capacity, nutrient mobilization, and modulation of host gene expression. When evaluated *in planta*, some of these strains improved water status, nutrient acquisition, antioxidant capacity, and symbiotic performance. However, the evidence remains fragmented: direct evidence in legumes under actual drought is limited to a few studies, mostly conducted under controlled conditions and with incomplete characterization of soil water status, whereas a large part of the literature relies on salinity, heat, or PEG-induced osmotic stress, non-legume hosts, or genomic and *in vitro* evidence. The available findings therefore establish plausible mechanisms and proof-of-concept results, but they do not yet demonstrate reliable field performance of extremophile-derived inoculants in legume production.

Future research should address four priorities. First, candidate strains should be characterized at the strain level, including genome sequencing, growth optima, and biosafety assessment, so that the extremophilic or extremotolerant status of each inoculant is documented rather than inferred from its habitat of origin. Second, drought experiments should follow standardized protocols that report soil water content or matric potential, plant water status, the duration and intensity of the deficit, and the phenological stage at which it is imposed, and should include well-watered controls. Third, multi-environment and multi-season field trials that encompass the complete crop cycle, or at least the reproductive stage (from anthesis to physiological seed maturity), are needed to evaluate yield, seed nutritional quality, and symbiotic performance, with detailed reporting of inoculant identity, formulation, viable-cell counts, persistence, and application protocols. Such approaches will be essential to determine whether the physiological, biochemical, and molecular responses observed under controlled conditions translate into consistent agronomic benefits under realistic agricultural environments.

Fourth, the ecological complexity of plant–inoculant interactions must be addressed explicitly. Future studies should integrate physiological, multi-omics, and microbiome approaches to identify the factors that govern inoculant establishment, persistence, and performance, including host genotype × strain interactions, soil type, and the composition of the native microbiota. Parallel testing of the same strains under drought, salinity, and heat in the same host would allow cross-stress transferability to be evaluated directly rather than inferred. Finally, functionally designed consortia that combine the stress tolerance of extreme-environment-derived strains with the rhizosphere competence of locally adapted bacteria represent a promising avenue for developing effective, resilient, and environmentally safe bioinoculants for legume production under increasing water scarcity.
